# Transforaminal Epidural Balloon Adhesiolysis via a Contralateral Interlaminar Retrograde Foraminal Approach: A Retrospective Analysis and Technical Considerations

**DOI:** 10.3390/jcm9040981

**Published:** 2020-04-01

**Authors:** Chan-Sik Kim, Yeon-Jin Moon, Jae Won Kim, Dong-Min Hyun, Shill Lee Son, Jin-Woo Shin, Doo-Hwan Kim, Seong-Soo Choi, Myong-Hwan Karm

**Affiliations:** 1 Department of Anesthesiology and Pain Medicine, Asan Medical Center, University of Ulsan College of Medicine, Seoul 05505, Korea; chrissky@naver.com (C.-S.K.); hdm27@hanmail.net (D.-M.H.); sjinwoo@hotmail.com (J.-W.S.); knaaddict@gmail.com (D.-H.K.); 2 Department of Anesthesiology and Pain Medicine, National Medical Center, Seoul 04564, Korea; yj.moon.ans@nmc.or.kr (Y.-J.M.); ssl8612@naver.com (S.L.S.); 3 Department of Anesthesiology and Pain Medicine, Eulji University Medical Center, Eulji University College of Medicine, Daejeon 35233, Korea; kjaewonee@naver.com; 4 Department of Dental Anesthesiology, Seoul National University Dental Hospital, Seoul 03080, Korea

**Keywords:** chronic pain, epidural balloon adhesiolysis, interlaminar, retrograde, lumbar spinal stenosis

## Abstract

Several treatment modalities have been proposed for foraminal stenosis, but the treatment options remain unsatisfactory. Previous studies have shown that transforaminal balloon adhesiolysis may be effective in patients with refractory lumbar foraminal stenosis. However, in patients with a high iliac crest, balloon catheter insertion may be difficult via a conventional transforaminal approach (particularly targeting the L5–S1 foramen). It has been reported that an epidural catheter can be placed easily by a contralateral interlaminar retrograde foraminal approach. Therefore, we applied this approach to L5–S1 transforaminal balloon adhesiolysis in patients with a high iliac crest. We retrospectively analyzed data from 22 patients who underwent combined epidural adhesiolysis and balloon decompression (balloon adhesiolysis) using the novel foraminal balloon catheter via a contralateral interlaminar retrograde foraminal approach. The pain intensity significantly decreased over the three-month period after balloon adhesiolysis (*p* < 0.001). There were no complications associated with the balloon procedure. The present study suggests that balloon adhesiolysis for L5-S1 foramen via a contralateral interlaminar retrograde foraminal approach may be an effective alternative for patients with a high iliac crest and refractory lumbar radicular pain due to lumbar foraminal stenosis. In addition, detailed procedural aspects are described here.

## 1. Introduction

Chronic lumbar radicular pain can adversely affect the quality of life. Transforaminal epidural steroid injection (TFESI) is one of the most common treatments for the management of chronic lumbar radicular pain, with or without low back pain (LBP), resulting from lumbar spinal stenosis, disc herniation, discogenic pain, and failed back surgery syndrome [1,2,3]. However, this approach is often only effective for a few weeks and may not improve patients’ disability [4]. Such patients may have epidural adhesions, which are an important cause of lumbar radicular pain with or without LBP [5,6,7]. Therefore, percutaneous epidural adhesiolysis is often used to remove epidural adhesions [8]; alternatively, combined epidural adhesiolysis and balloon decompression (balloon adhesiolysis) of the transforaminal epidural space can result in significant pain relief and functional improvement in patients with intractable lumbar radicular pain with or without LBP [9,10,11,12,13].

In patients with a high iliac crest, it may be difficult to insert an epidural catheter via a conventional transforaminal approach (particularly at L5–S1), although transforaminal epidural injection of drugs is still possible. Therefore, when inserting a catheter into the L5–S1 foramen in patients with a high iliac crest, foraminal direction should be considered. Previously, Jeong et al. showed that an epidural catheter can be placed easily at the ventrocaudal aspect of the existing nerve using a contralateral interlaminar retrograde foraminal (CIRF) approach [14]. Previous studies have shown that the contrast spreads well into the target nerve root and the ventral epidural space [14,15].

Therefore, we applied the CIRF approach to L5–S1 transforaminal balloon adhesiolysis in patients with a high iliac crest. This study retrospectively analyzed outcomes after foraminal balloon adhesiolysis via the CIRF approach in patients who have high iliac crest with intractable lumbar radicular pain with or without LBP. In addition, we describe the detailed procedure for this alternative approach for L5–S1 foraminal balloon adhesiolysis. 

## 2. Materials and Methods

This retrospective study was conducted at the pain clinic at Asan Medical Center, Seoul, Republic of Korea. The necessity for informed consent was waived as only recorded data were reviewed. We reviewed the electronic medical records of patients for all necessary data that were itemized and recorded at their visits to the pain clinic. The study protocol was reviewed and approved by the Ethical Committee of Asan Medical Center (approval number, 2018-1260), and the study was conducted in accordance with the Declaration of Helsinki.

### 2.1. Participants

**Figure 1:**
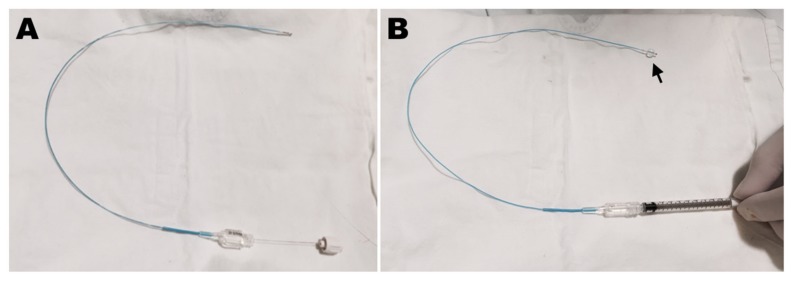
The novel inflatable balloon catheter used in the transforaminal approach (ZiNeuF^®^). (**A**) This 2-Fr inflatable balloon catheter is thinner than other catheters but is more maneuverable because of the guidewire. (**B**) The inflatable balloon is positioned on the tip of the catheter (arrow). After the air in the catheter is removed, the catheter is connected to a 1 mL Luer-Lock syringe containing 0.13 mL of contrast medium for ballooning.

The records of patients who underwent balloon adhesiolysis using the novel foraminal balloon catheter with guidewire (ZiNeuF^®^, JUVENUI, Seoul, Republic of Korea, Figure 1) in areas of L5–S1 foraminal stenosis via a CIRF approach in our institution between May 2016 and November 2018 were reviewed. All aspects of patient privacy and confidentiality were preserved.

The inclusion criteria for balloon adhesiolysis using a balloon catheter were as follows: (1) chronic (at least 3 months) L5–S1 foraminal stenosis patients aged ≥ 50 years; (2) lumbar radicular pain, with or without LBP, with an intensity ≥ 6 (out of 10) on the numerical rating scale (NRS); (3) patients unable to access the area of L5–S1 foraminal stenosis by conventional transforaminal technique because of the presence of a high iliac crest; (4) confirmed diagnosis of moderate or severe foraminal stenosis by magnetic resonance imaging (MRI) [16]; and (5) previous failure of conservative management, such as exercise therapy, physical therapy, analgesic medication, TFESI, or percutaneous epidural adhesiolysis with a balloon-less catheter. In all patients, previous treatment had either no effect or the effect did not last more than 3 months.

The exclusion criteria were as follows: (1) aged < 50 years; (2) acute pain for < 3 months; (3) could not exclude a confounding diagnosis of vascular disease or a disease of another origin; (4) signs of progressive neurological deficits or motor weakness; (5) allergies to steroids or contrast dyes; (6) uncontrollable or unstable opioid use; (7) coagulopathy; (8) systemic or injection site infection; (9) unstable medical or psychiatric condition; and (10) malignancy.

### 2.2. Interventional Technique

All patients underwent the same ballooning procedure in the area of foraminal stenosis by the methods described below. The balloon adhesiolysis procedure was performed by two well-trained pain specialists. The foraminal balloon catheter with guidewire was the same as that used in our previous study [17]. The intervention was conducted in an operating room that was prepared with emergency equipment and a single fluoroscopy C-arm system (OEC 9800; General Electric Healthcare, Little Chalfont, United Kingdom). After the patient entered the operating room, the patient was placed in the prone position. A pillow was placed under the abdomen to reduce the lordotic curvature of the lumbar spine. Pulse oximetry was monitored continuously, and noninvasive blood pressure was monitored every 5 min. The intervention was performed under sterile conditions, and betadine solution was used for skin preparation. 

#### 2.2.1. Insertion of the Guide Needle for a Contralateral Interlaminar Retrograde Foraminal Approach

**Figure 2:**
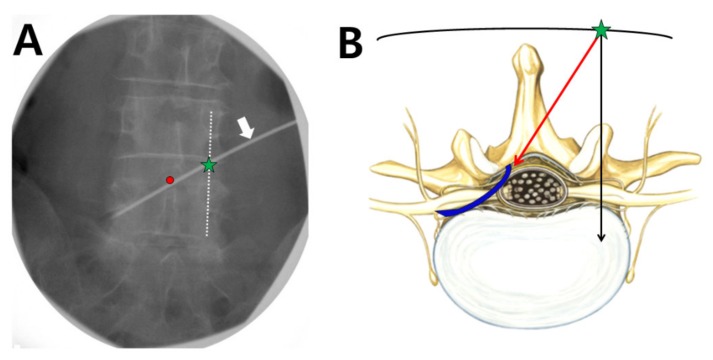
Determining the entry point and catheter pathway on the fluoroscopic anteroposterior view. (**A**) A simulated surface marker (white arrow) positioned between the lower portion of the ipsilateral pedicle, where the corresponding symptomatic spinal nerve exits, and the interlaminar epidural opening site over the interspinous process (red dot). The entry point of the guide needle (green star) is the point at which the contralateral longitudinal pedicular lines (dotted line) and the simulated surface marker intersect. (**B**) Schematic cross-sectional drawing of the route of the guide needle and foraminal balloon catheter. The red arrow shows the direction of the needle from the needle entry point (green star); this must cross the midline to ipsilateral epidural space, otherwise the catheter may be advanced into the contralateral neural foramen. The blue line shows the insertion pathway of the foraminal balloon catheter.

By using caudal tilt on the fluoroscopic view, we obtained a single contour of the upper endplate of the target vertebra. The target interlaminar space was checked in an anteroposterior fluoroscopic view. To simulate the guide needle and balloon catheter pathway, a surface marker (white arrow in Figure 2A) was positioned between the lower portion of the ipsilateral pedicle, where the corresponding symptomatic spinal nerve exits, and the interlaminar epidural opening site over the interspinous process (red dot in Figure 2A). The entry point of the guide needle was at the point where this simulated line and the contralateral longitudinal pedicular lines (dotted line in Figure 2A) intersected (green star in Figure 2A). Lidocaine (1%, 1 mL) was introduced at the entry point (green star in Figure 2B), and a 16-gauge guide needle was inserted and advanced in a medial direction (red arrow in Figure 2B) until it touched the bone at the spinous process. After it touched the bone, it was walked off and advanced across the midline to the ipsilateral epidural space using the ‘loss-of-resistance’ technique, and the needle tip was advanced to the point beyond midline to the ipsilateral side. When the needle was sufficiently advanced, the lateral and anteroposterior views were checked to confirm the accurate position of the needle. Similarly, access to the ipsilateral epidural space via a CIRF approach can be performed at the same level (i.e., L5–S1).

#### 2.2.2. Combined Epidural Adhesiolysis and Balloon Decompression via a Contralateral Interlaminar Retrograde Foraminal Approach

**Figure 3:**
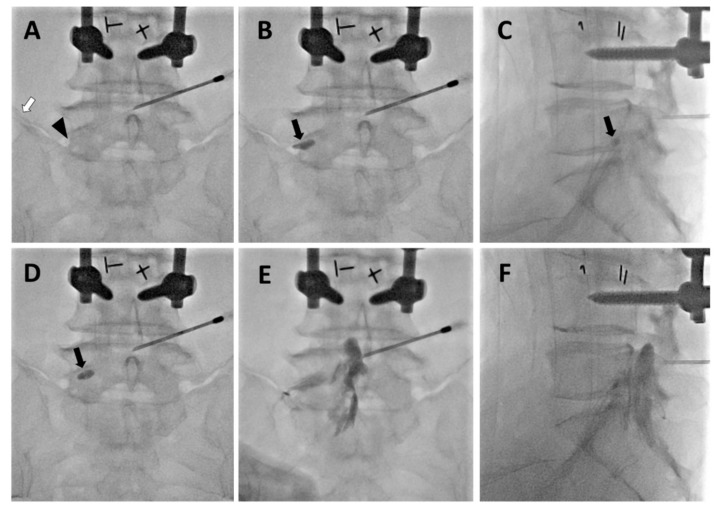
Serial fluoroscopic images of transforaminal balloon treatment via a retrograde interlaminar approach in patient H (see Table 1). (**A**) The foraminal balloon catheter with guidewire was inserted to the extraforaminal space through a guide needle, which was inserted via a retrograde interlaminar approach because of the patient’s high iliac crest (white arrow). The position of the foraminal balloon catheter can be checked with a fluoroscopic image of the guidewire (arrow head). (**B**) The foraminal balloon catheter was placed in the left L5–S1 intervertebral neural foramen. During ballooning with contrast medium, the grade of foraminal stenosis was visualized by the degree of balloon distortion (black arrow). (**C**) Lateral view in the state of Figure 3B. The inflated state of balloon in the intervertebral foramen is identified (black arrow). (**D**) Balloon treatment was performed by gradually pulling the catheter backward (black arrow). (**E**) After completion of the balloon procedure, the catheter was removed, and contrast medium was injected via the guide needle. The contrast medium is seen to spread well into the left L5–S1 intervertebral foramen and central epidural space. (**F**) Lateral view in the state of Figure 3E. The contrast medium is seen to spread well into the anterior and posterior epidural space.

After confirming that the needle was positioned in the ipsilateral epidural space, a 2-Fr foraminal balloon catheter containing a removable guidewire was inserted in a caudal direction. The balloon catheter was steered to the lower aspect of the pedicle into the ventral epidural space and the neural foramen with foraminal stenosis (blue line in Figure 2B and arrowhead in Figure 3A). After checking the position of the catheter tip (fluoroscopic anteroposterior and lateral view), the guidewire was removed. After the air in the catheter had been removed, the catheter was connected to a 1 mL Luer-Lock syringe (Becton-Dickinson, Franklin lakes, NJ, USA) containing 0.13 mL of contrast medium (Omnipaque^®^, iohexol, GE Healthcare, Oslo, Norway). If air remained in the balloon catheter, the radiographic image could have some air artifact, which can be mistaken for a stenotic lesion. As shown in Figure 3B,C, the balloon procedure was performed from the outlet of the neural foramen to the central epidural space by gradually pulling the catheter backward. Each ballooning period did not exceed 5 s to avoid the possibility of ischemic nerve damage. The ballooning pressure was controlled according to the grade of the patient’s pain. If moderate to severe pain was noted during balloon inflation, no further attempt was made for safety reasons. In the main target site of foraminal stenosis, the balloon was inflated 2 or 3 times without moving the catheter. The catheter was moved only in the balloon-deflated state. After completion of the balloon adhesiolysis procedure, the foraminal balloon catheter was removed in a deflated state. The contrast medium was injected via the guide needle to confirm effective spreading of the contrast dye from the central epidural space to the left extraforaminal space (Figure 3D). A mixture of 1% lidocaine, 5 mg of dexamethasone, and 1500 IU of hyaluronidase (total volume of 3 mL) was injected into the target area. After injection, the guide needle was removed and a simple dressing was applied.

In the recovery room, noninvasive blood pressure and pulse oximeter were monitored for 60 min. After confirmation that there were no procedure-related complications (e.g., unstable vital signs, dural puncture, and motor weakness), the patients were discharged.

### 2.3. Outcome Assessments and Follow-up

At the first visit (baseline), all patients were taught to use an 11-point NRS (0 = no pain and 10 = worst possible pain) to assess the intensity of lumbar radicular pain with or without LBP. The patients were followed up in the outpatient department of our pain clinic at 1, 3, and 6 months after the procedure. NRS pain scores were routinely obtained on follow-up. The data, including age, sex, height, weight, underlying disease, history of previous lumbar surgery, MRI finding, dermatome of radicular pain, complication of procedure, and additional procedure during follow-up periods were collected from electronic medical records. 

### 2.4. Statistical Analysis

Continuous variables are presented as means with standard deviation. Statistical analyses were conducted with repeated-measures analysis of variance using SPSS Statistics version 21 (SPSS, Inc., Chicago, IL, USA).

## 3. Results

In total, 22 patients underwent foraminal balloon adhesiolysis using the novel foraminal balloon catheter in areas of foraminal stenosis in our institution between May 2016 and November 2018 in accordance with the inclusion and exclusion criteria. These 22 patients were retrospectively analyzed in the present study, and the patients’ demographic characteristics are shown in Table 1. The mean age, height, weight, and body mass index (BMI) of patients were 69.45 ± 8.42 years, 160.04 ± 7.95 cm, 60.95 ± 10.76 kg, and 23.69 ± 3.00 kg/m^2^, respectively. In total, 12 (54.5%) patients had comorbidity or underlying disease. There were six (27.3%) patients with hypertension and five (22.7%) patients with diabetes mellitus (DM). Other diseases included hepatitis B virus liver cirrhosis, asthma, Raynaud syndrome, and coronary artery syndrome. Two (9.1%) patients had a past history of lumbar spine surgery; patient B underwent L3-5 laminectomy and discectomy, and patient H underwent L3–4 posterior lumbar interbody fusion (PLIF) surgery.

**Figure 4:**
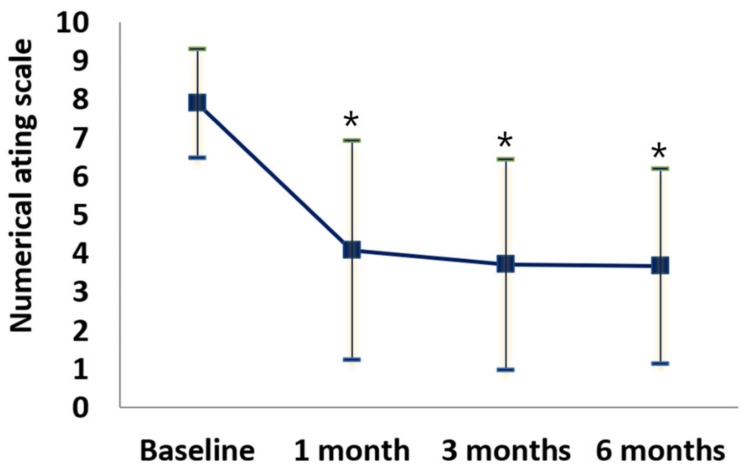
Numerical rating scale of lumbar radicular pain with or without lower back pain at baseline, 1, 3, and 6 months after combined epidural adhesiolysis and balloon decompression. * *p* < 0.001 versus baseline.

L5–S1 foraminal stenosis was found in all patients, and 17 (77.3%) patients also had lumbar central stenosis. The direction of symptoms was the left for 17 (77.3%) and right for 5 (22.7%) patients (Table 2). The mean baseline NRS of pain intensity was 7.91 ± 1.41, and the mean NRS pain score significantly decreased after one, three, and six months to 4.09 ± 2.84, 3.72 ± 2.73, and 3.68 ± 2.53, respectively (Figure 4). The NRS pain intensity improved over the six-month period after foraminal balloon adhesiolysis via a CIRF approach (*p* < 0.001). However, six (27.3%), six (27.3%), and four (18.2%) patients had no pain improvement (NRS < 2) at one, three, and six months after the balloon adhesiolysis procedure compared to baseline. The number of patients with minimally important (≥ 30% or ≥ two-point decrease of NRS) and significant changes (≥ 50% or ≥ four-point decrease of NRS) in pain intensity were 18 (81.8%) and 13 (59.1%) at six months, respectively.

There were no complications associated with the balloon procedure. During follow-up after balloon adhesiolysis, 7 (31.8%) patients underwent additional procedures. TFESIs were performed in four patients during the follow-up period. Two patients received medial branch blocks one month after the balloon procedure following pulsed radiofrequency (pRF) treatment to the medial branch three months after follow-up. One patient underwent TFESI one month after the balloon procedure and additional pRF to the L5 dorsal root ganglion because of continuation of symptoms (Table 3).

## 4. Discussion

Epidural adhesion can play a key role in the development of chronic lumbar radicular pain and/or LBP [5,6,18], and percutaneous epidural adhesiolysis is an effective way to alleviate the symptoms of these patients, particularly those who do not respond well to conventional epidural blocks [1,3,10,19]. As one of the methods of percutaneous epidural adhesiolysis, transforaminal balloon procedures can provide significant pain relief and functional improvement in patients with chronic intractable lumbar foraminal stenosis [11,13]. Distension of the epidural space by intermittent ballooning can lead to more effective mechanical detachment of perineural adhesion and achieve more efficient delivery of epidural injected drugs to the foraminal stenotic regions by increasing the marginal space of the stenotic area [10,11,12]. In Korea, the previously reported balloon-inflatable catheter (ZiNeu^®^, JUVENUI, Seoul, Korea) is commonly used for percutaneous epidural adhesiolysis via a caudal approach [9,10,12]. However, this catheter has a relatively large diameter, which can cause severe pain when entering the stenotic intervertebral foramen via a caudal approach. A recently developed novel foraminal balloon catheter designed specifically for the transforaminal approach is simple, soft, and less expensive (Figure 1). The diameter of the balloon catheter is smaller than that of the previous version, allowing entry into stenotic areas with limited pain. Moreover, the balloon catheter contains a guidewire that facilitates positioning in the stenotic foramen. However, in patients with a high iliac crest, balloon adhesiolysis via a conventional transforaminal approach can be challenging. In these patients, when the neural foramen is exposed at < 30 degrees on the fluoroscopic oblique view, the angle of guide needle insertion is too acute; as a result, the nerve root can be irritated causing pain during the procedure.

Previous studies suggested that the use of a retrograde interlaminar dorsal epidural approach with ventral epidural catheterization may be an effective approach to deliver medication to the target site [14,15,20]. Although the catheter is placed in the dorsal epidural space, contrast medium is delivered evenly to both the ventral and dorsal epidural spaces [14]. Similarly, as shown in the present study, a CIRF approach may be successfully adapted to foraminal balloon adhesiolysis in patients with radicular pain who are intractable to conventional treatment. The present data showed that foraminal balloon adhesiolysis via a CIRF approach significantly reduced lumbar radicular pain with or without LBP during a three-month follow-up period after procedures without complications. A CIRF approach for foraminal insertion of the balloon catheter can avoid the high iliac crest. Moreover, by passing through the central canal, the balloon catheter can reach the target neural foramen parallel to the nerve root. Importantly, after entry of the guide needle, its tip must be placed across the midline of the interlaminar space when the needle is entering the ipsilateral epidural space (Figure 3). If the tip enters the dorsal epidural space without crossing the midline, the balloon catheter may reach the contralateral side of the target area. All patients in the present study had a high iliac crest and were expected to be unsuitable for the conventional transforaminal approach for insertion of the balloon catheter. Therefore, we could place the guide needle in the epidural space using the interlaminar space with the CIRF approach. The balloon catheter was inserted in the neural foramen from the dorsal epidural space to the ventral epidural space near the symptomatic spinal nerve. In this way, we could overcome anatomical disadvantages and issues related to disease severity and insert the catheter more easily than with conventional transforaminal approaches. In all patients, the procedure was safely performed avoiding vascular and neural injury. However, these advanced techniques should be trained and practiced sufficiently, including a cadaver workshop before being performed on the patient.

Previous studies suggested that, in comparison with conventional TFESI, drugs can be delivered more effectively to the target site by retrograde interlaminar ventral epidural injection, usually at the ventral aspect of the nerve root sleeve, dorsal aspect of disc herniation, or stenotic lesion [14,20]. Moreover, in cases of dorsal epidural spread, the retrograde interlaminar approach showed greater flow than in the conventional transforaminal approach [14,20].

However, foraminal balloon adhesiolysis via the CIRF approach was less effective in five (22.7%) patients in this study. Patient C had no symptom improvement one month after the procedure and was additionally underwent TFESI. This might be because of DM neuropathy causing negative neuropathic symptoms. Patient H, who had previously undergone L3-4 PLIF, was not satisfied with symptom improvement and a TFESI was performed one month after follow-up; symptoms persisted at three months, and L5 pRF was conducted. Patient L did not respond at all for three months after the procedure because of DM neuropathy, and was subsequently treated with a small ketamine infusion. The symptoms of patient N also persisted, but the patient refused further interventional procedure and wanted to be treated with medication only. Patients R and S had minor symptom improvement and a medical branch block was additionally performed at one month after balloon adhesiolysis because the facet joint syndrome with axial pain became more pronounced during follow-up. After the diagnostic block confirmed co-existing facet joint syndrome, pRF of medial branch nerve was performed at the three month follow-up. These failed cases suggest that correct diagnosis and patient selection for balloon adhesiolysis is important. Indeed, previous reports have already demonstrated that factors associated with the outcome of balloon adhesiolysis for the management of chronic lumbar foraminal stenosis may be diabetes, combined LBP with lumbar radicular pain, degree of lumbar foraminal stenosis, the number of target levels, and a degenerative herniated intervertebral disc [10,13,21,22]. When considering balloon adhesiolysis, patients with DM neuropathy, failed back surgery syndrome, or axial pain should be selected cautiously. Taken together with the present findings, suggested indications of the foraminal balloon adhesiolysis via a CIRF approach are as follows: (1) patients unable to access L5–S1 foraminal stenosis by a conventional transforaminal technique because of the presence of a high iliac crest; (2) previous failure of conservative management, including TFESI for foraminal stenosis; and (3) patients without DM neuropathy or axial pain. 

This study had several limitations. First, this study was a retrospective study without a control group. This was not blinded, well-designed, or well-controlled. Second, two pain specialists treated all patients in this study, and some deviation of the described techniques may be present. Third, no other outcome measure tool except for pain intensity was used to assess the effectiveness of balloon adhesiolysis. Furthermore, the functional effect of the balloon adhesiolysis and medication effect were not considered in this study. Lastly, the analyzed sample size in the present study was small. This small sample size may be associated with no complications observed after the foraminal balloon adhesiolysis. More well-designed randomized controlled trials in the future will yield more reliable results about the effectiveness of this procedure.

## 5. Conclusions

In conclusion, the present study suggests that balloon adhesiolysis via a CIRF approach may be an effective alternative option for patients with a high iliac crest and refractory radicular pain due to lumbar foraminal stenosis.

## Figures and Tables

**Table 1:** Demographic characteristics of the study population.

Subject	Age	Sex	Height (cm)	Weight (kg)	BMI (kg/m^2^)	Underlying Disease	History of Lumbar Surgery
A	73	Male	166.8	63.7	22.9	None	None
B	74	Male	167.1	71.3	25.5	DM, asthma	L3–4–5 laminectomy
C	71	Male	168	80	28.3	DM, HTN	None
D	72	Male	165	53	19.5	Raynaud syndrome	None
E	64	Female	155	59	24.6	None	None
F	65	Female	152	48	20.8	None	None
G	68	Female	158	58	23.2	Hypothyroidism, angina	None
H	70	Female	153	55	23.5	None	L3–4 PLIF
I	58	Male	174	73	24.1	CAD,	None
J	79	Male	169	57	20.0	DM, asthma	None
K	80	Male	170	82	28.4	HTN, CAD	None
L	61	Male	166	74	26.9	DM, HTN	None
M	66	Male	158	70	28.0	None	None
N	73	Male	172	68	23.0	None	None
O	65	Male	155	53	22.1	DM, HBV LC	None
P	59	Female	155	52	21.6	None	None
Q	65	Male	160	60	23.4	None	None
R	76	Female	152	44	19.0	None	None
S	90	Male	155	53	22.1	HTN	None
T	64	Female	150	58	25.8	HTN	None
U	80	Female	150	45	20	HTN	None
V	55	Female	150	64	28.4	None	None

BMI: Body mass index, CAD: Coronary artery disease, DM: Diabetes mellitus, HBV LC: Hepatitis B virus liver cirrhosis, HTN: Hypertension, PLIF: Posterior lumbar interbody fusion.

**Table 2:** Procedural characteristics and outcomes of the study population.

Subject	Main Lumbar MRI Findings	Dermatome of Radicular Pain *	Pain Intensity (NRS)				Complication
			Baseline	1 M	3 M	6 M	
A	L4–5 central and Lt. L5 FS	Lt. L5 ^†^	8	3	2	2	None
B	L2–3 central and both L3,4,5 FS	Rt. L5	8	5	5	5	None
C	L2–3 central and both L3,4,5 FS	Lt. L5	7	7	7	7	None
D	L3–4 central and Rt. L4,5 FS	Rt. L5	8	6	6	5	None
E	L3–4–5 central and Lt. L5 FS	Lt. L5	10	0	0	0	None
F	Both L5 FS	Lt. L5	10	7	3	5	None
G	L4–5 central and both L5 FS	Lt. L5	6	3	3	2	None
H	L3–4 central and both L5 FS	Lt. L5	7	6	6	5	None
I	L3–4 central and Rt. L5 FS	Rt. L5	7	1	1	1	None
J	Both L5 FS	Lt. L5	10	0	0	0	None
K	L4–5 central and Lt. L5 FS	Lt. L5	8	3	2	4	None
L	both L3,4,5 FS	Lt. L5	10	10	10	10	None
M	L2–3–4–5 central and Lt. L4,5 FS	Lt. L5	8	4	4	3	None
N	L4–5 central and Rt. L5 FS	Lt. L5	6	5	5	5	None
O	L4–5 central and both L5 FS	Lt. L5	7	4	3	3	None
P	L4–5 central and Lt. L5 FS	Lt. L5 ^†^	10	2	2	2	None
Q	L2-3–4 central and Rt. L5 FS	Rt. L5	7	3	3	2	None
R	Severe scoliosis and Lt. L5 FS	Lt. L5	8	8	5	5	None
S	L3–4 central and Rt. L4,5 FS	Rt. L5	9	8	8	7	None
T	Lt. L4,5 FS	Lt. L5	8	1	1	3	None
U	L4–5–S1 central and both L3,5 FS	Lt. L5 ^†^	6	4	6	5	None
V	L3–4–5–S1 central and Lt. L5 FS	Lt. L5	6	0	0	0	None

FS: Foraminal stenosis, Lt.: Left, M: Month, MRI: Magnetic resonance imaging, NRS: Numerical rating scale, Rt.: Right. * Indicates the mainly targeted nerve root (e.g., L5 means lumbar 5 nerve root located in the L5–S1 foramen). ^†^ Contralateral interlaminar retrograde approach at the same level (i.e., L5–S1) was performed in these patients.

**Table 3:** Additional interventional procedures in the 6-month follow-up period for the study population.

Additional Procedures	Patients (*n*, %)
None	15 (68.2)
TFESI *	4 (18.2)
L5 pRF *	2 (9.1)
MBB, MB pRF	2 (9.1)

During follow-up after the balloon adhesiolysis, 7 (31.8%) patients underwent additional procedures. MB: Medial branch, MBB: Medial branch block, pRF: Pulsed radiofrequency, TFESI: Transforaminal epidural steroid injection. * One patient, H, received pRF after the TFESI.
